# Heterologous Expression of Two Jatropha Aquaporins Imparts Drought and Salt Tolerance and Improves Seed Viability in Transgenic *Arabidopsis thaliana*

**DOI:** 10.1371/journal.pone.0128866

**Published:** 2015-06-12

**Authors:** Kasim Khan, Pallavi Agarwal, Arti Shanware, Vidhu Aniruddha Sane

**Affiliations:** 1 Plant Gene Expression Lab CSIR-National Botanical Research Institute (Council of Scientific and Industrial Research), Lucknow-226001, India; 2 Rajiv Gandhi Bio-Technology Centre, R.T.M.N.U., Nagpur, India; National Institute of Plant Genome Research, INDIA

## Abstract

Drought and high salinity are environmental conditions that cause adverse effects on the growth and productivity of crops. Aquaporins are small integral membrane proteins that belong to the family of the major intrinsic proteins (MIPs), with members in animals, plants and microbes, where they facilitate the transport of water and/or small neutral solutes thereby affecting water balance. In this study we characterized two aquaporin genes namely, plasma membrane intrinsic protein (*PIP2;7*) and tonoplast intrinsic protein *TIP1;3* from *Jatropha curcas* that are localised to the plasma membrane and vacuole respectively. Transgenic *Arabidopsis thaliana* lines over-expressing *JcPIP2;7* and *JcTIP1;3* under a constitutive promoter show improved germination under high salt and mannitol compared to control seeds. These transgenic plants also show increased root length under abiotic stress conditions compared to wild type Col-0 plants. Transgenic lines exposed to drought conditions by withholding water for 20 days, were able to withstand water stress and attained normal growth after re-watering unlike control plants which could not survive. Transgenic lines also had better seed yield than control under salt stress. Importantly, seed viability of transgenic plants grown under high salt concentration was 35%-45% compared to less than 5% for control seeds obtained from plants growing under salt stress. The effect of *JcPIP2;7* and *JcTIP1;3* on improving germination and seed viability in drought and salinity make these important candidates for genetic manipulation of plants for growth in saline soils.

## Introduction

One of the causes of reduced agricultural productivity is high soil salinity as most crops are sensitive to salinity. Although irrigation helps in productivity in arid and semi-arid areas, over-exploitation of irrigation schemes and wrong practices lead to salinization of soil. According to various reports 20% of all irrigated lands world-wide (equivalent to 62 million ha) are salt affected with some estimates being as high as 50%. This amounts to approximately US$ 27.3 billion loss due to salt-induced land degradation in irrigated areas [1]. In India, according to 2011report of WHO, 60% of land area is under agriculture of which 35% is irrigated. Salinity affects 8.56 million ha of land in India and is responsible for reduced yields in crops.

Water relations in plants are very important to maintain various physiological processes. A tight control is exerted over water loss through stomata under favourable as well as unfavourable conditions as photosynthesis has to be balanced against water loss for survival under unfavourable conditions [2–4] Regulation of water movement across cellular membranes is regulated by a family of water channel proteins called aquaporins. This family has been shown to facilitate the efficient transport of water molecules as well as small solutes across animal and plant membranes [5–9]. These belong to a highly conserved membrane protein family called major intrinsic protein (MIP) [10–12]. Plant aquaporins consists of a large family with 35, 33, 28 and 71 homologues in Arabidopsis, rice, grapes and cotton respectively [13–17].

Based on sequence homology and cellular localization studies, plant aquaporins are divided into four subgroups. Sub family 1 comprises of plasma membrane intrinsic proteins (PIP) (further divided in two subgroups, PIP1 and PIP2) whereas the TIP sub-family consists of members that are targeted to the tonoplast. PIPs and TIPs are the most abundant aquaporins in the plasma membrane and vacuolar membrane (tonoplast), respectively [13–14]. The third subfamily comprises the nodulin- 26–like intrinsic membrane proteins (NIPs), while the fourth class comprises small basic intrinsic proteins (SIPs) [18]. Aquaporins are expressed in almost all tissues and are required in organ development, root and shoot water uptake, maintenance of cell turgidity as well as in response to abiotic stresses [19]. Their role in abiotic stresses has attracted attention since many aquaporins members show differential expression in response to abiotic stresses such as drought, salinity and cold with some being activated and others suppressed [15, 20–23]. Over-expression analysis of aquaporins have provided contrasting results with some aquaporins such as those from maize, rice and wheat providing stress tolerance and others making plants more sensitive to abiotic stresses [23–25].

*Jatropha curcas* (family *Euphorbiaceae*) is widely distributed in tropical and subtropical parts of the world and is promoted as a renewable biofuel due to high seed oil content. It also has other potential uses in medicinal and cosmetics industry and also as a land reclamation plant because of its ability to withstand water stress. Due to its hardiness the plant can potentially be tapped as a source of genes for withstanding water stress as well as for improved oil biosynthesis. In this study we have identified two aquaporins in a screen for genes related to seed development and oil biosynthesis from seed specific library and show that these are important water channel proteins in providing abiotic stress tolerance.

## Materials and Methods

### Plant material, growth conditions and treatments

Developing seeds of *Jatropha curcas* (Acc. no. NBRI-UA-Alm-0406), growing in the CSIR-NBRI field, were used for RNA isolation. Seeds of stages 1–7 [26] as well as other tissues like flowers, leaves, stem and seed coat were frozen in liquid nitrogen and kept at -70°C until further use. Jatropha plants were grown at 28–30°C; 16 h/8 h day/night photo-period conditions for 1 month and the subjected to drought treatment by withholding irrigation for 20 days. A separate set of plants were grown in normal well watered conditions for the same time as control. For salt stress, young Jatropha leaves were excised and their petioles dipped in water (as control) or 200 mM NaCl for salt uptake for different time intervals. Leaf samples from control and various drought and salt treated plants were collected and frozen in liquid nitrogen and stored at -70°C

Seeds of Col-0 (WT *Arabidopsis thaliana*) and lines over-expressing *JcPIP2;7* and *JcTIP1;3* were soaked in water at 4°C for 2 days prior to sowing in soilrite. Pots were placed in culture room maintained at 20–22°C; 16 h/8 h day/night photo-period and plants were watered, supplemented with nutrients at regular intervals.

### RNA extraction, cDNA preparation and cloning of Jatropha aquaporins

Total RNA was extracted from various tissues and samples described aboveaccording to the method described by Singh et al [27]. First strand cDNA (prepared using MMLV reverse transcriptase, Fermentas) from various samples was pooled and used as template to amplify the full length ORF of *JcPIP2;7* and *JcTIP1;3* using gene specific primers (S1 Table) on a Bio-Rad PCR machine (California, USA). Amplified gene fragments of *JcPIP2;7* and *JcTIP1;3* were cloned in pTZ57R/T (Fermentas), sequenced, analyzed using BLAST tools and compared with other known plant aquaporin sequences.

### Sequence analysis

A comparative analysis of *JcPIP2;7* and *JcTIP1;3* with known sequences was carried out using NCBI Blast server [28]. The Clustal W program [29] was used for sequence alignment with other Jatopha aquaporin sequences. WoLF PSORT: Protein Sub cellular localization prediction was used to generate a PostScript output from aligned sequences. Phylogenetic analyses were conducted using MEGA version 6 by using bootstrap values of 500 data sets [30].

### Transient expression in onion epidermis

The full-length coding sequences of *JcPIP2;7* and *JcTIP1;3* were cloned upstream of the coding sequence for the Green Fluorescence Protein (GFP) under the control of the CaMV35S promoter in the vector pBI121-GFP. These aquaporin genes and empty vector pBI121-GFP were co-expressed with the plasma membrane m-cherry marker pm-rk and tonoplast m-cherry marker vac-rk (http://www.bio.utk.edu/cellbiol/markers/)[31]. Biolistic transformation of onion (*Allium cepa L*.) epidermal cells was performed with 1.6 μm gold particles (coated with 1μg of plasmid DNA/transformation) at 1100 psi helium pressure with the help of Bio-Rad gene gun (California USA). After incubation at 24°C for 24 h, the sub-cellular localization of expression in terms of fluorescence was observed using confocal laser scanning microscopy (LSM510 META; Carl Zeiss, 20X, Heidelberg, Germany)

### Expression analysis by Semi-quantitative RT-PCR and real time qRT PCR

The relative expression levels of *JcPIP2;7* and *JcTIP1;3* in different tissues of *Jatropha curcas* were analyzed using semi-quantitative reverse transcription polymerase chain reaction (RT-PCR). 35 cycles were performed with each cycle consisting of denaturation at 94°C for 30 s, annealing at 55°C for 10 s, and extension at 72°C for 30 s using gene specific primers (S1 Table) followed by a final extension of 7 min at 72°C. Real-time PCR analysis was carried out using SYBR green PCR mix (Fermentas). The Jatropha actin gene was used as reference and water samples were taken as calibrator.

### Expression of aquaporins in yeast mutant

The *JcPIP2;7* and *JcTIP1;3* ORFs were cloned in the yeast expression vector pUG-35 at *XbaI* and *SacI* sites for functional validation. The yeast strain 10560-6B [MATa leu2::hisG trp1::hisG his3::hisG ura3-52 aqy1::KanMX4 aqy2::HIS3; derivative of *Saccharomyces cerevisiae* (aqy-null)] was used in this work. The plasmids pUG35*Jc*PIP2;7 (VASP2;7), pUG35*Jc*TIP1;3 (VAST1;3) and the control empty vector (VAS0) were transformed into *S*. *cerevisiae* (aqy-null strain) by the lithium acetate method according to Yeast Transformation Kit manual (Sigma). Transformants were selected on YNB medium without uracil (strains and constructs are listed in S2 Table). The strains grown under salt stress were assessed on solid YNB medium supplemented with NaCl (75 and 100 mM). Multiple 10 fold serial dilutions of the original culture were prepared and plates were inoculated with 5 μl culture drops and incubated at 30°C. Growth was observed after 3–5 days.

### Generation of transgenic Arabidopsis lines over-expressing *JcPIP2;7* and *JcTIP1;3*

The ORFs of *JcPIP2;7* and *JcTIP1;3* were cloned in pBI121 (Clontech) at *XbaI-SacI* sites and Agrobacteria (GV3101) containing these constructs were used to transform Arabidopsis (ecotype Columbia) plants by the Agrobacterium mediated floral dip method [32–33]. Transgenic seeds were screened on kanamycin and confirmed by PCR and then grown to homozygous T3 generation. Three independent homozygous lines for each transgene were used for further analysis.

### Germination assays

Seeds were stratified at 4°C for 48 h and then transferred to 22°C. For germination analysis, 50 seeds from each line were sown on half MS agar medium or MS medium supplemented with Mannitol (350 mM) and NaCl (150, 200 mM). The germination percentage was determined at different time points. For root length measurements, the Arabidopsis seeds were grown on half MS agar medium containing different Mannitol (350 mM) and NaCl (150 mM) concentrations. The plates were positioned vertically for the evaluation of root growth.

#### Drought stress treatments and relative water content measurements for transgenic lines

For the drought experiments, seeds were sown and germinated into a 5-cm pot filled with 200 g soilrite and grown for 15 days under well watered condition. After that water was withheld for an additional 20 days. The plants were then irrigated regularly to check the recovery process in control and transgenic plants. After 15 days of drought exposure, relative water content (RWC) of the plants was measured in leaves of WT and transgenic lines. Turgid weight (TW) of plants was measured after soaking for 4 h in distilled water at room temperature under constant light. Prior to soaking plants, fresh weight (FW) was recorded. Total dry weight (DW) was recorded after drying these plants at 70°C to a constant weight. RWC was calculated using the following expression:

RWC = (FW – DW) / (TW - DW)×100.

## Results

### The Jatropha aquaporin family contains 22 members

During the course of our studies related to seed development and oil biosynthesis in Jatropha [26], we identified two genes encoding aquaporins from a seed specific cDNA library. These were selected for further characterization. Since aquaporins belong to a large family, we also analysed the aquaporin gene family in *Jatropha* using the Jatropha Genomic sequence database.

Twenty two putative aquaporin genes from Jatropha were identified from the Jatropha genome database (http://www.kazusa.or.jp/jatropha/) [34]. The 22 putative aquaporin polypeptide sequences encoded by these genes could be classified under various MIP (major intrinsic protein) subfamilies. Five aquaporins were found to belong to the TIP subfamily (3 TIP1 and two other TIPs), while nine belonged to the PIP sub-family (3 PIP1 and 6 PIP2). The remaining eight belonged to SIP and NIP sub-families. The genes were named as per the Arabidopsis nomenclature based on sequence homology to Arabidopsis genes. Table 1 provides a list of the putative aquaporins present in the Jatropha genome (http://www.kazusa.or.jp/jatropha/), their size, sub-cellular localization and number of exons and introns. The three members of the PIP1 subgroup (PIP1;2, PIP1;3 and PIP1;4) were predicted to encode polypeptides of 264, 212 and 287 amino acids respectively whereas the six PIP2 group members ranged in size from 267 to 284 amino acids with the exception of PIP2;5 (Jcr4S22721.20) which was larger and encoded a putative protein of 505 amino acids. It appears that there is gene merger in PIP2;5. The PIP1 and PIP2 groups showed 63–81% identity with each other. All nine PIP members possessed conserved dual NPA amino acid motifs (for water permeation). Except for PIP2;5 which had ten transmembrane helices (TMH), the other PIPs had five to six TMHs. Eight of the nine PIP protein sequences were predicted to be localized to the plasma membrane (WoLF PSORT programme) while one PIP i.e., JcPIP1;3 was predicted to be localized to the cytoplasm.

**Table 1:** Putative aquaporins present in Jatropha.

Gene ID	Gene name	Length (CDS/AA/Genomic)	Genomic organization exon/ Intron	TMH	NPA	sub-cellular localization	Orthologoues in *Arabidopsis thaliana*
Jcr4S09885.10	JcPIP1;2	795/264/1163	4/3	5	2	PM	PIP1;2 (AT2G45960.1)
Jcr4S00014.190	JcPIP1;3	639/212/1581	5/4	5	2	CYTO	PIP1;3 (AT1G01620.1)
Jcr4S00097.120	JcPIP1;4	864/287/1482	2/1	6	2	PM	PIP1;4 (AT4G00430.1)
Jcr4S01535.30	JcPIP1;5	972/323/1540	7/6	6	2	CYTO	PIP1;5 (AT4G23400.1)
Jcr4S05069.20	JcPIP2;1	849/282/3195	4/3	6	2	PM	PIP2;1 (AT3G53420.2)
Jcr4S01797.60	JcPIP2;2	855/284/1131	4/3	6	2	PM	PIP2;2 (AT2G37170.1)
Jcr4S01824.20	JcPIP2;4	804/267/2090	4/3	5	2	PM	PIP2;4 (AT5G60660.1)
Jcr4S22721.20	JcPIP2;5	1518/505/3934	6/5	10	2	PM	PIP2;5 (AT3G54820.1)
Jcr4S02148.40	**JcPIP2;7**	843/280/1280	4/3	6		PM	PIP2;7 (AT4G35100.1)
Jcr4S05761.40	JcPIP2;8	852/283/3188	4/3	6	2	PM	PIP2;8 (AT2G16850.1)
Jcr4S02015.50	JcTIP1;1	678/225/1150	4/3	4	1	VC	TIP1;1 (AT2G36830.1)
Jcr4S09350.30	JcTIP1;2	759/252/854	2/1	6	2	V	TIP1;2 (AT3G26520.1)
Jcr4S16114.20	**JcTIP1;3**	759/252/1289	3/2	6	2	VC	TIP1;3 (AT4G01470.1)
Jcr4S05795.10	JcTIP1;4	705/234/1011	2/1	4	1	PM	TIP1;1(AT2G36830.1)
Jcr4S00458.120	JcTIP2;1	747/248/926	3/2	6	2	VC	TIP2;1 (AT3G16240.1)
Jcr4S01015.30	JcTIP5;1	759/252/969	3/2	6	2	CYTO	TIP5;1 (AT3G47440.1)
Jcr4S01385.20	JcTIP4;1	744/247/1041	3/2	6	2	CYTO	TIP4;1 (AT2G25810.1)
Jcr4S01391.80	Jc β-TIP	774/257/927	3/2	6	2	CYTO	β-TIP (AT1G17810.1)
Jcr4S05431.20	JcTIP2;2	753/250/1292	3/2	6	2	V	TIP2;2 (AT4G17340.1)
Jcr4S00197.140	JcNIP1;2	747/248/1797	5/4	4	2	PM	NIP1;2 (AT4G18910.1)
Jcr4S10731.10	JcNIP3;1	834/277/1235	5/4	6	2	PM	NIP3;1 (AT1G31885.1)
Jcr4S02066.20	JcNIP1;1	570/189/1347	4/3	4	1	VC	NIP1;1 (AT4G19030.1)
Jcr4S07404.30	JcNIP4;1	843/280/1272	5/4	5	2	VC	NIP4;1 (AT5G37810.1)
Jcr4S00877.40	JcNIP4;2	876/291/2281	5/4	5	2	PMC	NIP4;2 (AT5G37820.1)
Jcr4S01286.80	JcNIP5;1	897/298/2803	4/3	5	1	PM	NIP5;1 (AT4G10380.1)
Jcr4S00555.10	JcNIP6;1	870/289/2255	6/5	5	0	PM	NIP6;1 (AT1G80760.1)
Jcr4S02777.30	JcNIP7;1	1662/553/2979	5/4	5	3	PM	NIP7;1 (AT3G06100.1)
Jcr4S21380.20	JcSIP1;1	708/235/708	1/0	5	1	VC	SIP1;1 (AT3G04090.1)
Jcr4S05789.30	JcSIP1;2	318/105/318	1/0	2	0	VC	SIP1;2 (AT5G18290.1)
Jcr4S13597.20	JcSIP2;1	369/122/958	2/1	2	0	CYTO	SIP2;1 (AT3G56950.2)

Aquaporins used for characterization in this study are underlined.

The five TIP family members in Jatropha ranged in size from 225–261 amino acids and shared 57–80% identity with one another. Three of the TIPs contained six TMHs while JcTIP1;1 and JcTIP4;1 had 4TMHs. JcTIP1;1 and JcTIP4;1 had only one NPA motif while the other three TIPs had two NPA motifs commonly found in TIPs from other plants. All the TIPs were predicted to be localized to the tonoplast except for TIP3;1 (PM localization).

The NIP sub family members in Jatropha were present in six sub groups with members of the family showing a minimum of 30% identity within subgroups. They ranged in size from 189 to 551 amino acids. The NPA motifs were less conserved in this family unlike the PIP and TIP sub-family with the Ala being replaced sometimes by Ser/Thr or Val.

**Fig 1:**
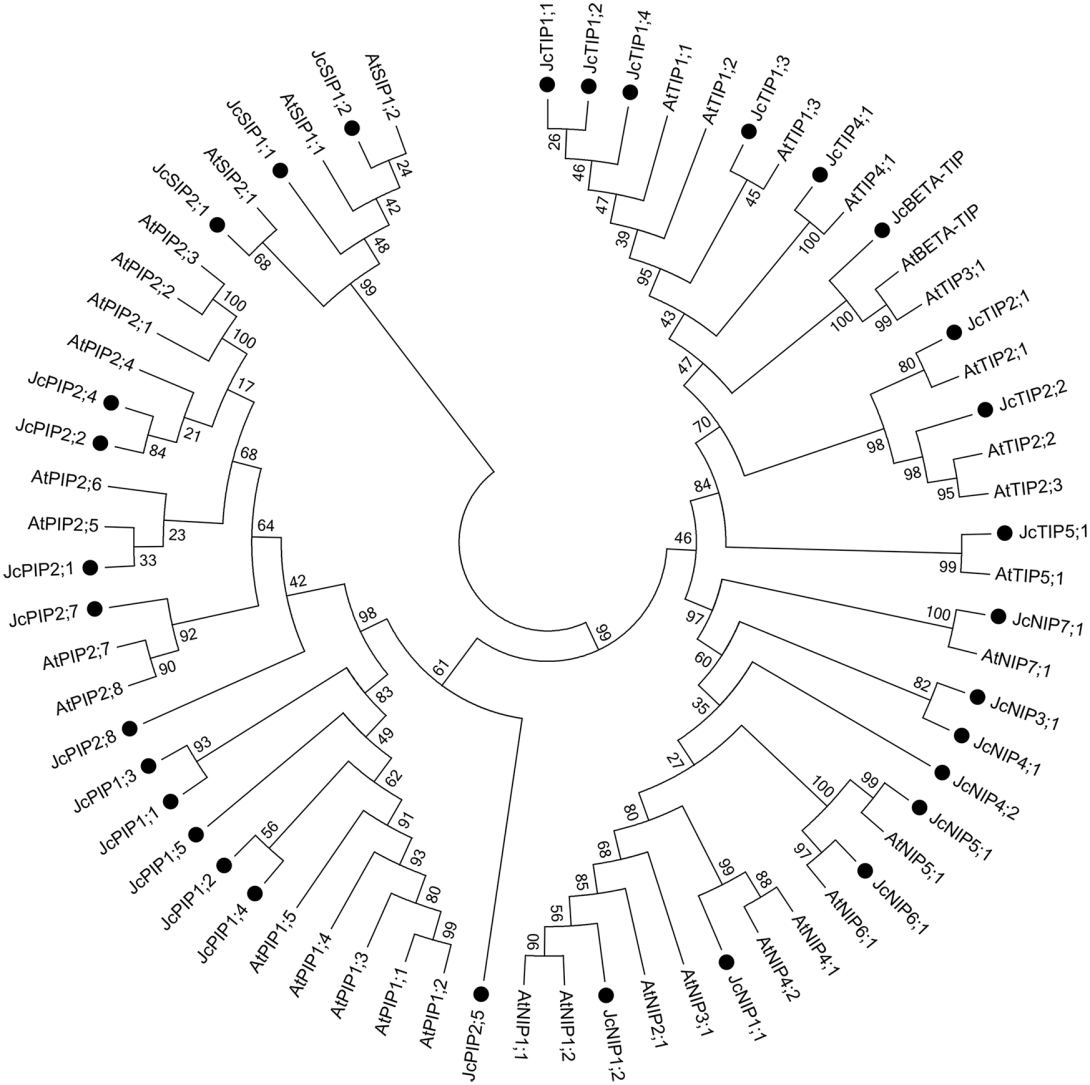
Phylogenetic analyses of members of the Jatropha aquaporin family with members of Arabidopsis. Sequence relationship of putative *Jatropha* aquaporin protein sequences with *Arabidopsis* to establish nomenclature to unknown genes. All the Arabidopsis sequences were obtained from TAIR (http://www.arabidopsis.org/) and *Jatropha* aquaporins were retrieved from the Jatropha genome database (http://www.kazusa.or.jp/jatropha/). The NJ (neighbor-joining method) tree was constructed by Mega 6 program using bootstrap method.

SIP sub-family members were present in only two sub groups SIP1 and SIP2. The predicted polypeptide length ranged from 105 to 235 amino acids and like NIPs, SIPs shared low sequence identity which ranged from 17%-40%. A phylogenetic tree was constructed using Jatropha and Arabidopsis sequences to establish the similarity between Arabidopsis and Jatropha genes. (Fig 1). The phylogeny shows the distinct grouping of the Jatropha and Arabiodpsis TIP, NIP, PIP1, PIP2 and SIP families.

### Identification and subcellular localization of *JcPIP2;7* and *JcTIP1;3*

To date, only two PIP members (*JcPIP1* and *JcPIP2* renamed as *JcPIP1;3* and *JcPIP2;7* in this manuscript) have been reported in Jatropha [35] and none have been functionally characterized. During screening of the Jatropha seed specific cDNA library, we obtained two aquaporin genes belonging to PIP2 and TIP1 sub-families that were identical to *JcPIP2;7* (accession no. ABM54183) and *JcTIP1;3* (accession no. HQ222607). These aquaporins are predicted to be localized to the plasma membrane and the tonoplast respectively.

**Fig 2:**
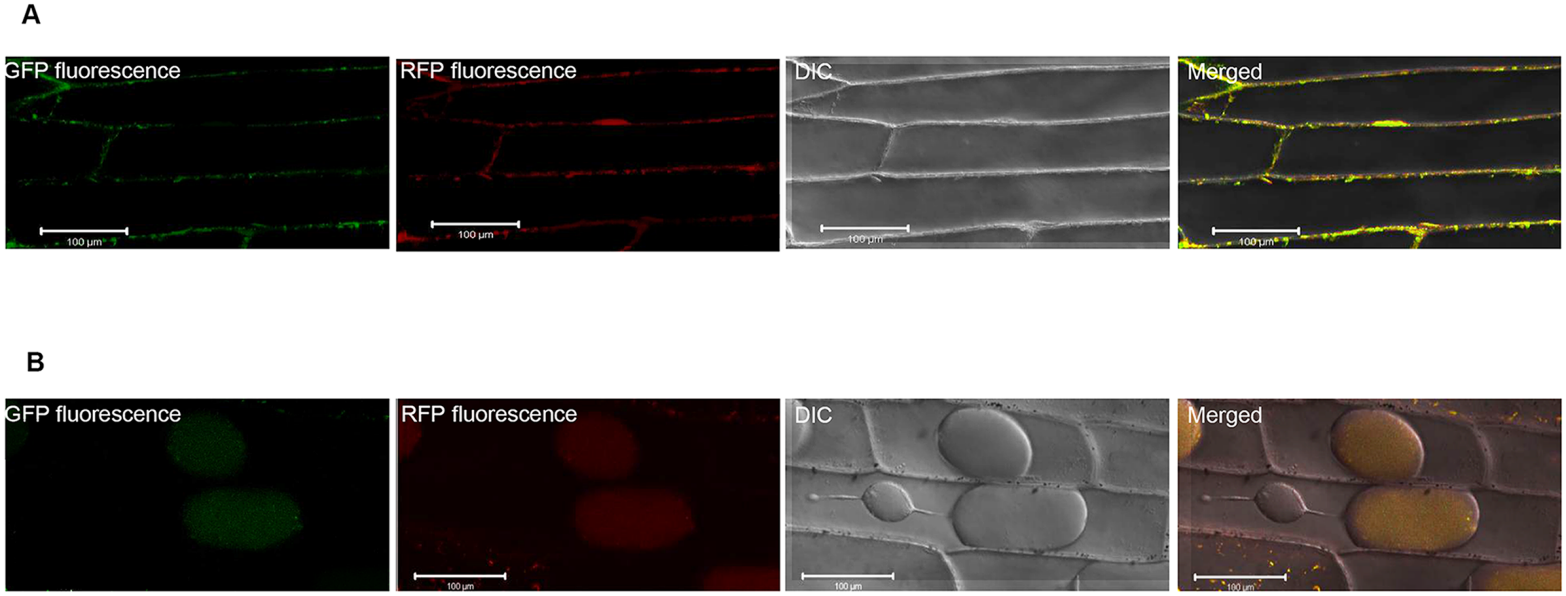
Sub-cellular localization of *JcPIP2;7*::*GFP* and *JcTIP1;3*::*GFP* proteins in onion epidermal cells. Onion epidermal cells were transiently co-transformed with **(A)** JcPIP2;7::GFP with pm-rk (plasma membrane marker) and **(B)** JcTIP1;3::GFP with vac-rk (vacuolar marker). The merged image shows localization of JcPIP2;7::GFP to the plasma membrane and that of JcTIP1;3::GFP to the vacuole/tonoplast. Scale bar = 100 μm.

To verify the sequence based prediction of sub-cellular location of *JcPIP2;7* and *JcTIP1;3*, constructs containing the cDNA encoding the green fluorescent protein were fused in frame to the aquaporin cDNA and transiently co-expressed with plasma membrane (pm-rk) and tonoplastic (vac-rk) markers in onion peel. Fluorescence results suggested that *JcPIP2;7* was mainly localized to the plasma membrane whereas *JcTIP1;3* was found to be localized in the tonoplast and vacuole (Fig 2A and 2B). The vacuolar localization could however be an artifact considering that the tonoplast marker m-cherry vac-rk was also localized in the vacuole instead of only tonoplast.

### Tissue and seed specific expression patterns of *JcPIP2;7* and *JcTIP1;3*

**Fig 3:**
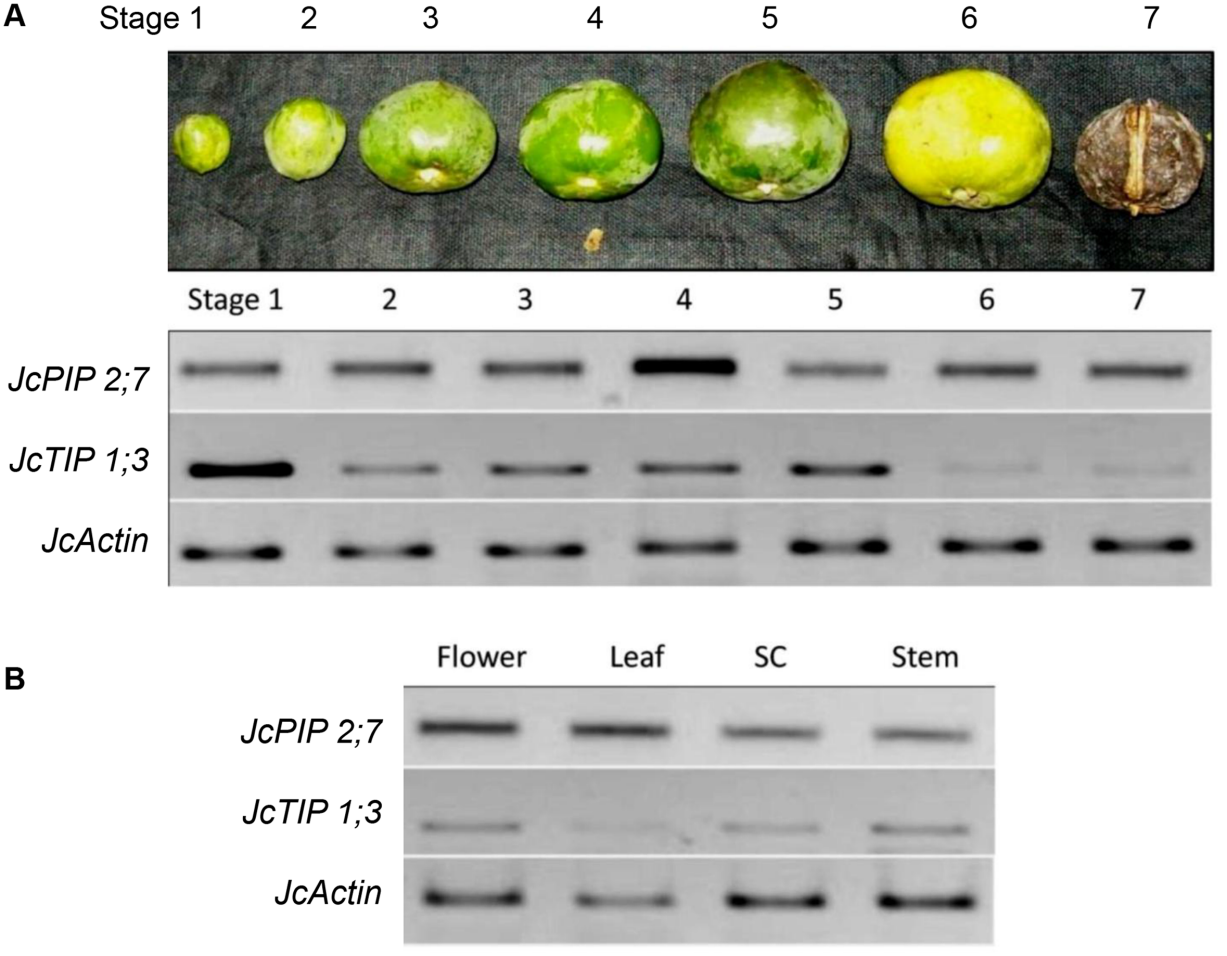
Transcript patterns of Jatropha aquaporins in developing seeds and vegetative tissues. (A) Transcript levels of *JcPIP2;7* and *JcTIP1;3* in different stages of seed development (1–7); (B) mRNA abundance of *JcPIP2;7* and *JcTIP1;3* in flower, leaf, seed coat and stem tissues of Jatropha by semi-quantitative RT PCR. Jatropha actin was used as the reference gene.

We next studied the transcript accumulation patterns of *JcPIP2;7* and *JcTIP1;3* in seeds and other tissues using semi-quantitative PCR. Both genes showed differential transcript accumulation in seeds and other vegetative tissues (Fig 3A and 3B). *JcPIP2;7* was transcribed in all developing stages of Jatropha seeds with maximum transcript accumulation in stage IV. *JcTIP1;3* had the highest expression in early stages of seed development. Levels of *JcTIP1;3* transcripts decreased with seed maturation with very low expression in mature seeds (Fig 3A). Both the genes expressed in leaves, flowers and stem although the levels of *JcTIP1;3* were comparatively lower than *JcPIP2;7* in the corresponding tissues. Transcripts of *JcPIP2;7* accumulated more in leaves and flowers than in stem and seed shells (Fig 3B).

### Transcript accumulation of *JcPIP2;7* and *JcTIP1;3* during abiotic stresses

**Fig 4:**
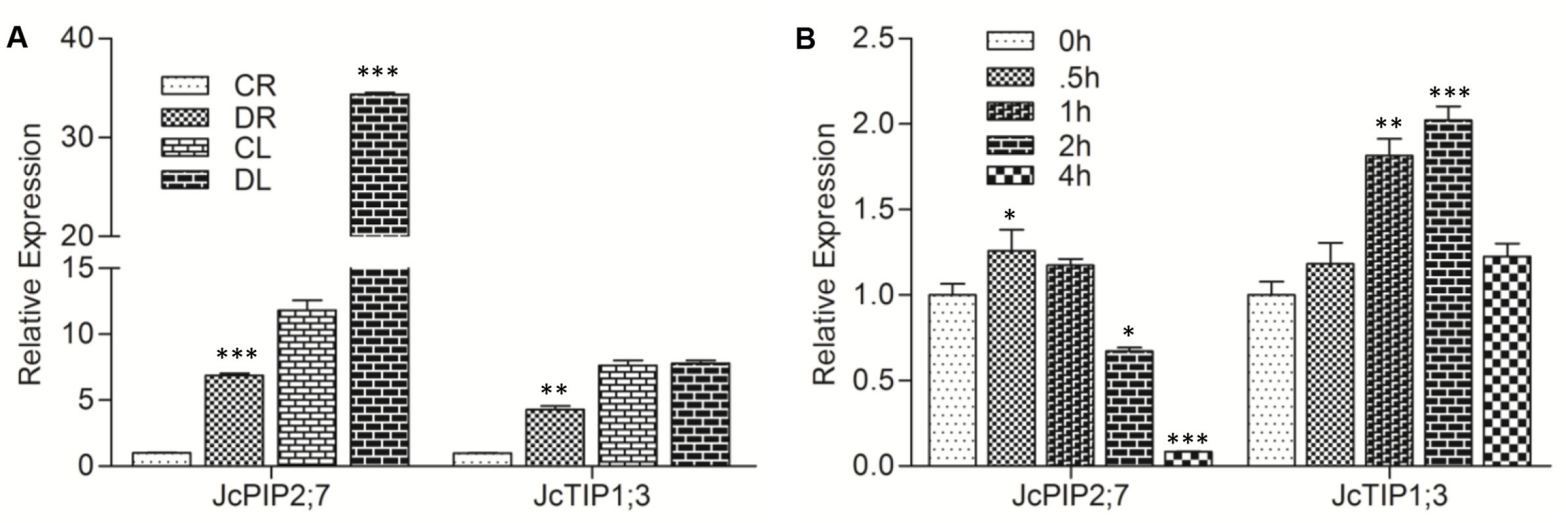
Expression of *JcPIP2;7* and *JcTIP1;3* in Jatropha under drought and salt stress. **(A)** Relative expression of *JcPIP2;7* and *JcTIP1;3* in control roots (CR), drought treated roots, (DR), control leaves (CL) and drought treated leaves (DL) of Jatropha. Jatropha plants were grown in well watered conditions for 1 month and then subjected to drought treatment by withholding irrigation for 20 days along with a control as described in material and methods. **(B)** Relative qPCR expression of *JcPIP2;7* and *JcTIP1;3* in young Jatropha leaves, treated with 200mM NaCl. Jatropha actin gene was used as reference gene and water samples were taken as calibrator. Bars show mean and error bars denote the standard deviation values.

Since aquaporins have been shown to play role in stress tolerance, we checked the expression of *JcPIP2;7* and *JcTIP1;3* in leaves and roots of *Jatropha* after exposure of plants to water stress and salt stress. In general, both, *JcPIP2;7* and *JcTIP1;3* showed an 8–10 folds higher expression in leaves than roots under control conditions (Fig 4A). Water stress resulted in seven folds higher induction of *JcPIP2;7* and a four folds higher induction of *JcTIP1;3* in drought exposed roots compared to control tissues. A three folds increase in transcript levels of *JcPIP2;7* was also observed in drought exposed leaves. However, no change was seen in leaves for *JcTIP1;3* (Fig 4A).

Treatment of leaves with NaCl (200 mM) did not result in any significant change of *JcPIP2;7* transcript levels in Jatropha leaves for up to 1h but thereafter there was a decline in its transcript levels to about a tenth of the control. In contrast, *JcTIP1;3* expression was induced by about two folds post 1 h salt treatment (Fig 4B).

### Complementation of aquaporins in yeast mutant

In order to check if these two putative aquaporins are functional, we examined whether these aquaporins could restore the growth of the yeast aqui null strain, YSH1172 (S2 table) under salt stress. The mutant strain did not express yeast specific aquaporins. The *JcPIP2;7* and *JcTIP1;3* genes were expressed in the yeast strain under the control of METα promoter. As shown (S1 Fig) the mutant strain transformed with *JcPIP2;7*, *JcTIP1;3* and empty vector could grow on YNB media. In the presence of 75 mM NaCl, *JcPIP2;7* transformed cells were able to grow even at 10^–4^ dilution, whereas the mutant strain transformed with the empty vector could not. At 100 mM NaCl, neither was able to grow at 10^–4^ dilution. Similarly transformation with *JcTIP1;3* allowed the cells to grow at a dilution of 10^–3^ at 100 mM NaCl unlike cells transformed with empty vector (S1 Fig).

### Over expression of *JcPIP2;7* and *JcTIP1;3* enhances salt and drought tolerance in transgenic *Arabidopsis thaliana*

#### Effect on seed germination

**Fig 5:**
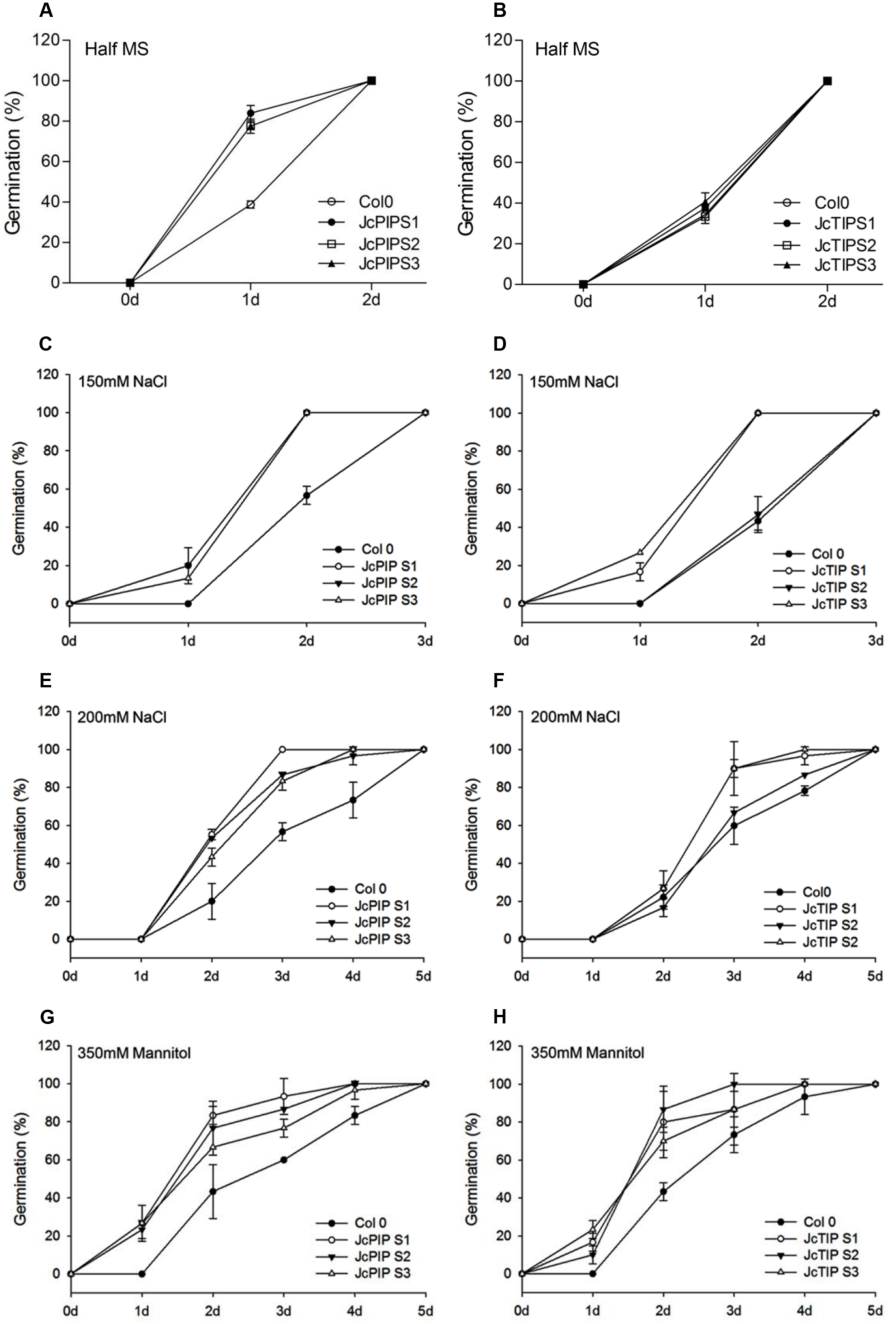
Effect of salt and mannitol stress on seed germination in lines expressing *JcPIP2;7* and *JcTIP1;3*. Germination of wild type Arabidopsis and transgenic seeds expressing *JcPIP2;7* and *JcTIP1;3* was monitored in presence or absence of different concentrations of salt and mannitol. **(A&B)** Percent germination of the wild type Col0 and transgenic Arabidopsis seeds expressing *JcPIP2;*(A) and *JcTIP1;3* (B) on half MS medium. **(C&E)** Percent germination of seeds of lines expressing *JcPIP2;7* in presence of 150 (C) and 200 mM (E) NaCl. **(D&F)** Percent seed germination in seeds of lines expressing *JcTIP1;3* in presence of 150 (D) and 200 mM (F) NaCl. **(G&H)** Percent seed germination lines expressing *JcPIP2;7* (G) and *JcTIP1;3* (H) in presence of 350 mM mannitol. Values and means were obtained from three independent experiments. Dots on lines are mean of percent germination and error bars represent ±SD.

In order to test the functionality of the two genes, transgenic Arabidopsis lines over-expressing *JcPIP2;7* and *JcTIP1;3* under constitutive CaMV35S promoter were developed and three independent homozygous lines for each gene were used for studying their growth parameters under unstressed and stressed conditions. On half MS, in absence of any stress, the seeds of transgenic lines expressing *JcPIP2;7* showed early germination compared to control (untransformed) seeds with a germination percentage of 75–80% after 24 h compared to less than 40% germination in case of control (Fig 5A). No differences were observed in germination of transgenic lines expressing *JcTIP1;3* with respect to control (Fig 5B). Since ABA negatively regulates seed germination, the germination pattern was also checked in presence of 1 μm ABA. As shown in S2 Fig, lines expressing *JcPIP2;7* did not show any difference in germination in presence of ABA. However, the ABA dependent inhibition of germination was reduced in lines over-expressing *JcTIP1;3* resulting in 60–80% germination in transgenic lines on day 2 compared to just 30% germination in control.

Seeds of these transgenic lines over-expressing the two different aquaporins also germinated early under saline stress conditions. On 150 mM NaCl, transgenic seeds over-expressing *JcPIP2;7* showed 15–20% germination after 24 h and 100% germination after 48 h (Fig 5C). Control seeds on the other hand were unable to germinate in 24h in presence of 150 mM NaCl and showed only 56% germination after 48 h and complete germination on day 3. A higher salt concentration (200 mM NaCl) exerted a more severe stress on seeds of Col-0 leading to only 50% germination after 72 h and complete germination after 5 days (Fig 5E). On the other hand, *JcPIP2;7* over-expressing seeds could tolerate high salinity stress and showed 45–50% seed germination after 48 h and 100% germination after just 72 h. Similarly, seeds of two of the three lines expressing *JcTIP1;3* (lines 1 and 3) germinated completely within 48 h in the presence of 150 mM NaCl and within 72 h on 200 mM NaCl, while the third behaved like the control (Fig 5D and 5F, S3 Fig).

On agar plates containing 350 mM mannitol, no control seed could germinate after 24 h as against 20–25% germination in all the three *JcPIP2;7* lines. By day 3, 75–90% transgenic and 55% control seeds germinated while complete germination occurred by day 4 in transgenic lines and day 5 in control (Fig 5G). Seeds of different lines over-expressing *JcTIP1;3* also tolerated high mannitol stress with 70–90% germination after 48h compared to 40% germination in controls. Complete germination in controls was only seen on day 5 compared to day 3 and 4 in transgenic lines. (Fig 5H, S3 Fig-).

#### Effect on root length

**Fig 6:**
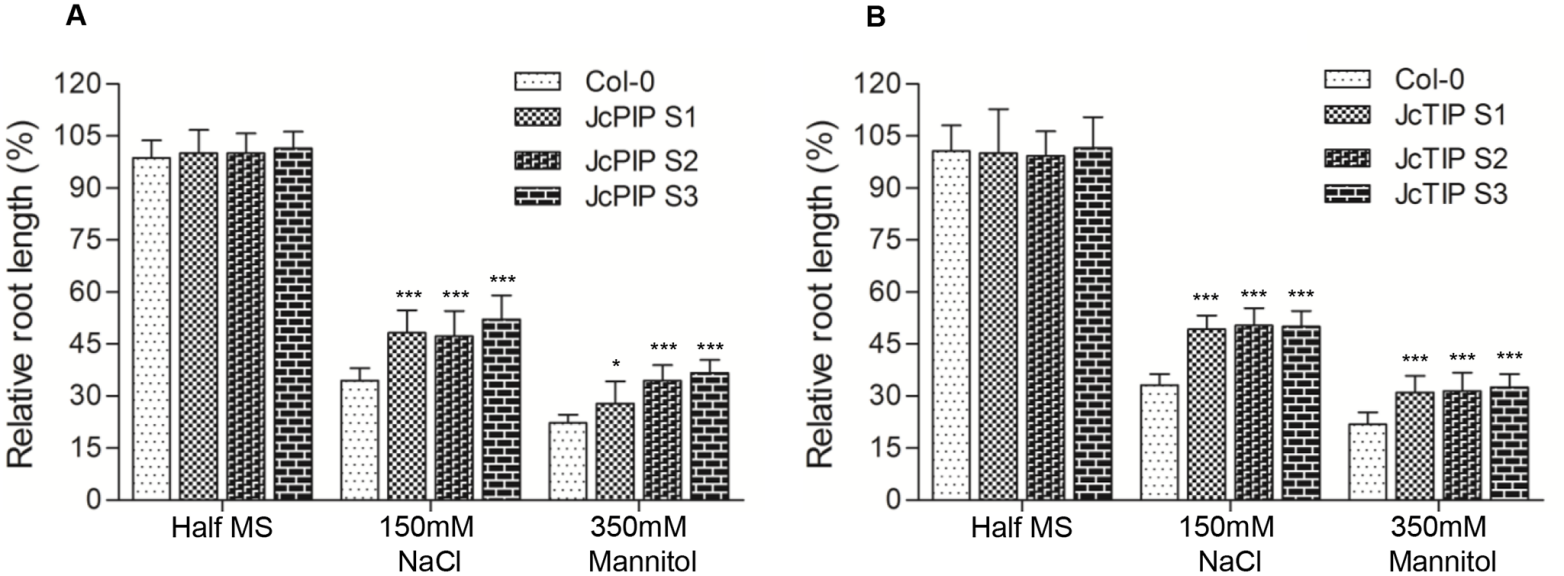
Relative root growth of transgenic seedlings expressing *JcPIP2;7* and *JcTIP1;3* under salt and mannitol stress. **(A)** Relative percent root length of WT Col-0 and transgenic seedlings expressing *JcPIP2;7* in presence of 150 mM NaCl and 350 mM mannitol. **(B)** Relative percent root length of WT Col-0 and transgenic seedlings expressing *JcTIP1;3* in presence of 150 mM NaCl and 350 mM mannitol. Seeds of transgenic lines along with wild type Col-0 were grown vertically in agar plates containing NaCl or mannitol. Root length was measured in 7 days old seedlings. For relative root length, root length of each line in different stress conditions was compared to root length in half MS medium (taken as 100%). Bars are mean of root length percent of each line and error bars represent ±SD. Asterisks indicate a significant differences at *P<0.05, **P<0.01, ***P<0.001 with respect to corresponding controls.

Apart from germination, abiotic stresses also affect other aspects of plant development such as root growth. To study these, primary root growth of transgenic seedlings was measured under high salt and mannitol conditions. On half MS medium, root growth of transgenic seedlings over-expressing *JcPIP2;7* and *JcTIP1;3* was similar to Col-0 seedlings (Fig 6A and 6B). When grown in presence of 150 mM NaCl root growth of both control and transgenic *JcPIP2;7* lines was reduced. However, transgenic lines expressing *JcPIP2;7* showed a comparatively reduced root inhibition in presence of NaCl than in control. Compared to a reduction of 65–70% in NaCl grown roots of control plants, transgenic plants showed a reduction of only 45–50%. Like NaCl, mannitol treatment (350 mM) inhibited primary root growth in Arabidopsis seedlings to just 22% of control in half MS. In contrast, root length in different *JcPIP2;7* expressing lines was reduced to 30–37% of that in half MS (Fig 6A). Roots of transgenic lines over-expressing *JcPIP2;7* were thus about 50-% longer in 150 mM NaCl and 350 mM mannitol compared to controls suggesting that root growth in transgenic lines was more tolerant to osmotic stresses.

Lines expressing *JcTIP 1;3* also showed reduced root length inhibition compared to control in 150 mM NaCl and 350 mM mannitol (Fig 6B). While NaCl treatment inhibited control roots to just 35% of the length of unstressed conditions, plants expressing *JcTIP1;3* were inhibited to 50–52% of the unstressed values. Treatment with 350 mM mannitol inhibited control roots to 25% of the unstressed values compared to 30–32% in *JcTIP1;3* expressing roots suggesting that lines expressing JcTIP1;3 were also relatively more tolerant to abiotic stresses than controls.

### Expression of stress related genes in transgenic lines expressing *JcPIP2;7* and *JcTIP1;3* under control and salt stress

In order to decipher the possible role of *JcPIP2;7* and *JcTIP1;3* in response to salt stress, the expression of some ABA induced stress-related marker genes such as *RD22*, *RD29A* and *RD29B* was analyzed in transgenic lines and wild type. Expression of all these genes were induced by salt treatment in control plants as reported earlier [36]. Transgenic lines over-expressing *JcPIP2;7* had only marginally higher expression of these stress marker genes under control unstressed conditions (S4 Fig). Under salt treatment, the changes in expression levels of endogenous *RD22* and *RD29A* genes in *JcPIP2;7* over-expressing plants did not show a consistent pattern in the two independent lines and appeared similar to control. In lines expressing *JcTIP1;3* however, two of the stress marker genes *RD29A* and *RD29B* showed 2–3 fold higher transcript levels under unstressed as well as salt stressed conditions compared to Col-0 plants (S4 Fig-). These data suggest that *JcPIP2;7* and *JcTIP1;3* interact differently with the abiotic stress signal machinery.

### Transgenic plants perform better under stress conditions

**Fig 7:**
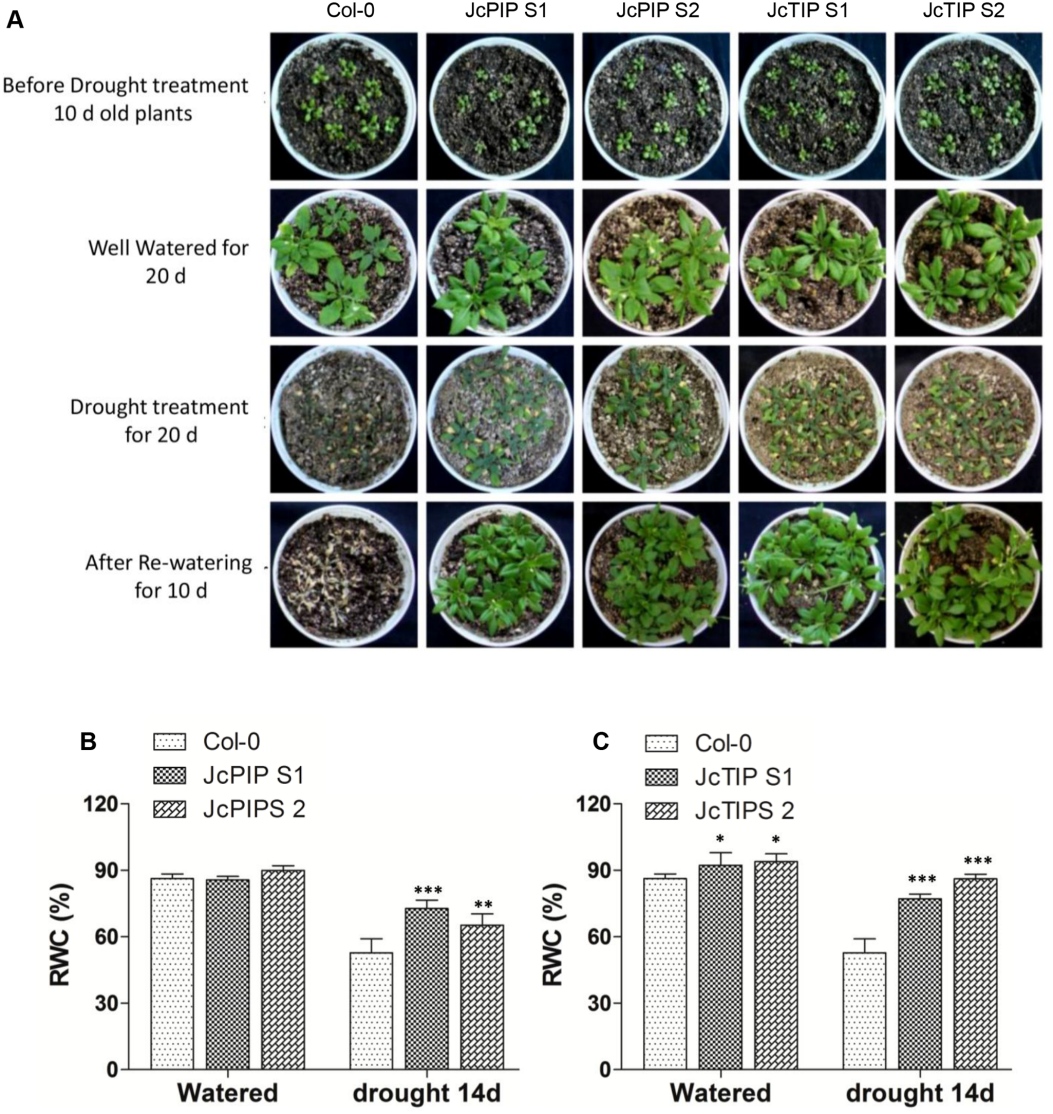
Performance of transgenic Arabidopsis plants expressing *JcPIP2;7* and *JcTIP1;3* upon exposure to drought stress. (A) Photograph of potted plants of WT Col-0 and transgenic Arabidopsis lines over-expressing *JcPIP2;7* and *JcTIP1;3* subjected to drought stress by withholding irrigation. Drought stress treatment was imposed on 10 day old plants for next 20 days. (B, C) Relative water content (RWC %) in WT Col-0 and transgenic plants over-expressing *JcPIP2;7* and *JcTIP1;3* respectively before and after 15 days of drought stress. Bars show mean while error bars denote the standard deviation values. Asterisks indicate a significant difference at *P<0.05, **P<0.01,***P<0.001 with respect to corresponding controls.

Our results related to root growth in high mannitol and salt showed that transgenic plants over-expressing aquaporins are more tolerant to osmotic stresses. So, we further tested the performance of plants over-expressing *JcPIP2;7* and *JcTIP1;3* during drought stress. Control Col-0 plants and transgenic lines over-expressing *JcPIP2;7* and *JcTIP1;3* were grown in well watered condition for 10 days and then subjected to drought. After 15 days of drought treatment, Col-0 plants showed wilting and growth retardation whereas plants of different lines over-expressing *JcPIP2;7* and *JcTIP1;3* were green and did not show wilting unlike in control plants. To examine the survival rates, plants were held under water deficit condition for an additional five days and then re-watered. Control plants, post 20 days drought, were severely damaged (Fig 7A). In contrast, although growth in transgenic plants was considerably reduced, the plants were nevertheless green and much larger than controls. During the recovery phase following re-watering, control plants could not revive whereas all plants of transgenic lines over-expressing *JcPIP2;7* and *JcTIP1;3* survived and regained growth. This improved drought tolerance of the transgenic plants was also correlated with higher % relative water content data. After 15 days of drought stress, %RWC of leaves from lines over-expressing *JcPIP2;7* and *JcTIP1;3* was 16% and 28% higher than Col-0 plants (Fig 7B and 7C).

**Fig 8:**
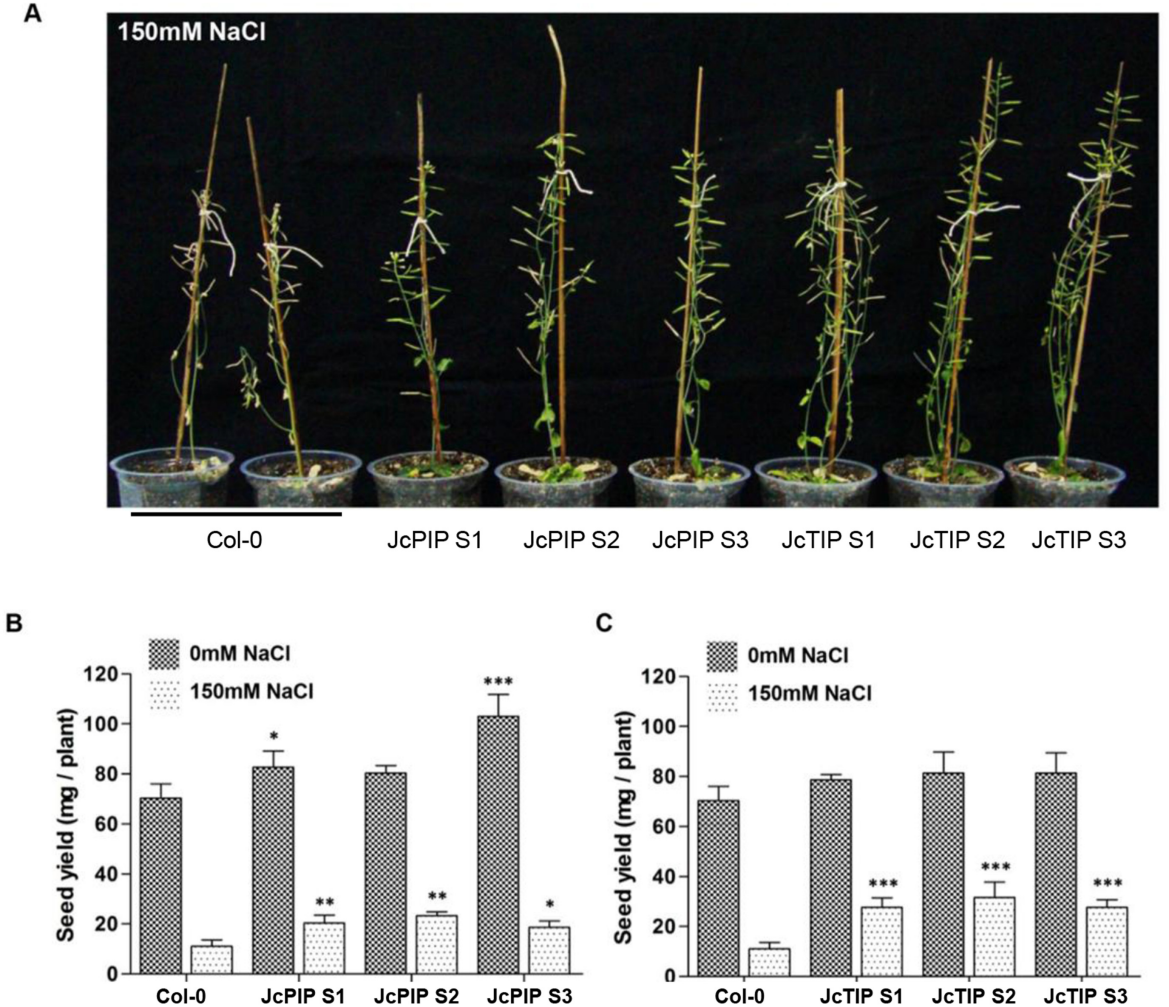
Performance of transgenic Arabidopsis plants expressing *JcPIP2;7* and *JcTIP1;3* upon exposure to salt stress. (A) Photograph of Wild-type Col-0 and transgenic plants over-expressing *JcPIP2;7* and *JcTIP1;3* grown in normal conditions in soilrite. After 10 days the plants were treated with and without 150 mM NaCl for the entire duration of their life cycle. (B, C). Total seed yield in WT Col-0 and transgenic plants over-expressing *JcPIP2;7* (B) and *JcTIP1;3* (C) in presence and absence of 150 mM NaCl. Values represent mean ±SD (n = 6) for each line and asterisks indicate a significant difference at *P<0.05, **P<0.01,***P<0.001 with respect to corresponding controls.

Plants of transgenic lines and wild type Arabidopsis were also grown in presence of 150 mM NaCl to study their comparative performance under salt stress. As shown in Fig 8A transgenic lines were less sensitive to salt stress and showed better growth. Total seed yield per plant under normal conditions was on average 22% higher in all *JcPIP2;7* lines as compared to control (Fig 8B). In the presence of 150 mM NaCl the seed yield was reduced to one sixth in control. The reduction was much lower in *JcPIP2;7* lines with the transgenic lines yielding 45% higher compared to control grown under similar stress conditions. Lines expressing *JcTIP1;3* also performed better than controls under salt stress with seed yield being three fold higher than control plants growing under saline stress conditions (Fig 8C).

**Fig 9:**
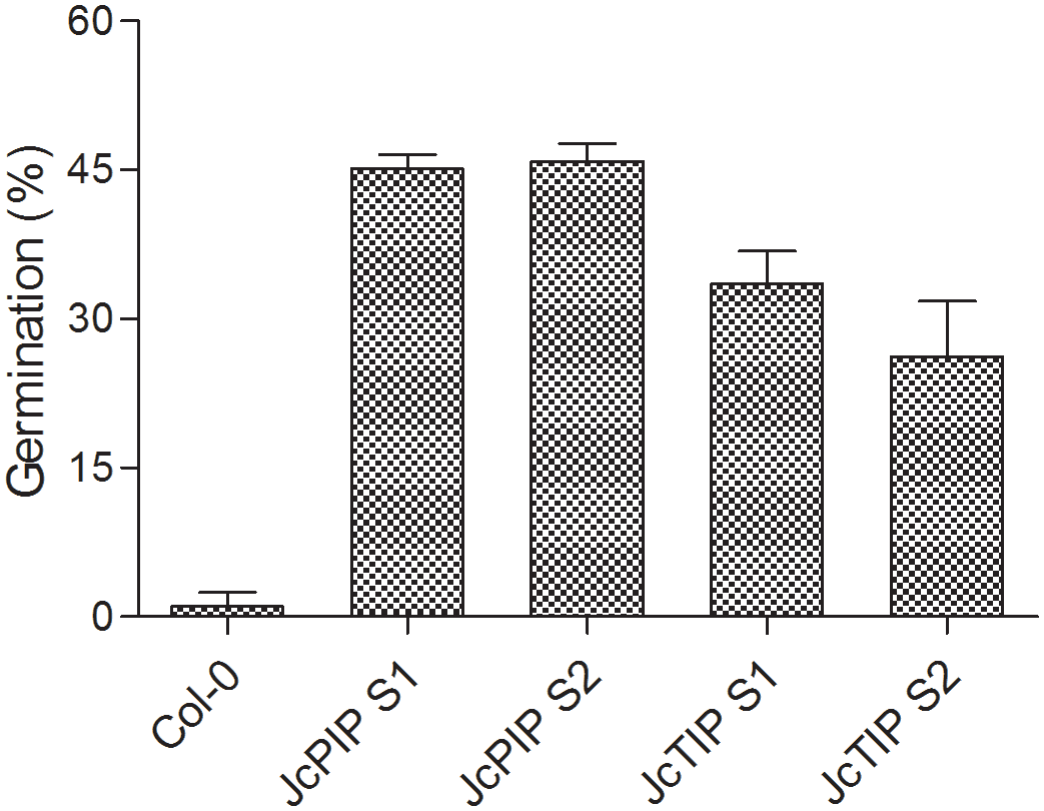
Percent germination of seeds obtained from plants of Col-0 and transgenic lines expressing *JcPIP2;7* and *JcTIP1;3* (grown in presence of 150mM NaCl) on half MS plates. JcPIP S1 and JcPIP S2 are homozygous lines expressing *JcPIP2;7* while JcTIP S1 and JcTIP S2 are homozygous lines expressing *JcTIP1;3*.

We next checked the viability of seeds obtained from plants growing under salt stress. Surprisingly, although both control and transgenic plants set seeds, the seeds of salt grown control plants showed poor viability with less than 3% germination. In contrast, seeds of transgenic lines over-expressing *JcPIP2;7* grown in presence of salt showed 45% viability (germination) on half MS plates. Viability of seeds of lines expressing *JcTIP1;3* obtained from growth under salt stress was also far better than control (35% germination) although less than the *JcPIP2;7* lines. (Fig 9). Thus expression of the two aquaporin genes not only improved seed yield under saline stress but had a more profound effect on seed viability of plants grown on salt stress.

## Discussion

Abiotic stress tolerance (towards drought, salt or cold) in plants requires efficient control over water balance to allow physiological activities to continue in the face of stress. Aquaporins are a multi-gene family of proteins that facilitate water and solute movement across different tissues throughout development and growth. These are not only required for growth and increase in organ size but also for maintenance or suppression of hydraulic conductance during stress and recovery after stress [37–38]. Accordingly, the expression levels of various members of the large aquaporin family have been known to be both positively as well as negatively regulated in osmotic stresses depending upon the need for increasing or decreasing hydraulic conductance. Members of all classes namely PIP, TIP, NIP and SIP have been shown to differentially regulate such responses although the structural or functional basis behind those that promote and those that suppress hydraulic conductance is not yet clear. With global increase in temperatures, drier monsoons and salinization of irrigated soils, reduction in yields due to osmotic stresses is becoming a major problem that needs to be overcome. Identification of genes that can be beneficial under mild to moderate stresses and enable better growth and yield under such conditions is a challenge.

*Jatropha* is a relatively drought tolerant crop that can survive water stress and can potentially yield better candidate genes for efficient stress tolerance. In this study, we have identified two aquaporin genes from *Jatropha* namely, *JcPIP2;7* and *JcTIP1;3* and performed detailed functional analysis in transgenic Arabidopsis at three developmental stages: a) germination, b) root growth in seedlings and c) whole plant growth and yield under abiotic stress conditions. Through these studies we show that both these genes could be useful for growth under moderate water and salt stresses. These genes, although identified from a seed-specific library of *Jatropha*, are also expressed rapidly in response to drought stress especially in roots with expression increasing almost three folds in both cases. This suggests that their expression might be governed by dehydration cues in seed as well as during water/osmotic stress. Both genes are able to partly complement the yeast aquaporin mutant (aqy null strain), in mild salt stress allowing growth up to 75 mM NaCl (PIP2;7) and 100 mM NaCl (TIP1;3).

Constitutive expression of these two aquaporins in Arabidopsis leads to improved and faster seed germination under water and salt stress conditions most likely due to reduced inhibition of water imbibition under stress. This is an observation that has also been noted upon expression of *PgTIP1*, *BnPIP1*, *BnTIP2* and the rice *OsPIP1;1* and *OsPIP2;7* genes that accelerate seed germination [39–41]. Nevertheless, there are distinct phenotypic differences between *JcPIP2;7* and *JcTIP1;3* expressing seeds with respect to their germination in absence of stress and in presence of ABA. Plants expressing *JcPIP2;7* show improved germination even in absence of any stress unlike *JcTIP1;3* which behaved similar to controls. One likely explanation for the differences in absence of stress could lie in the location of the aquaporins. In the plasma membrane where water permeability is limited by a factor of almost 100 compared to tonoplast [42], over-expression of *JcPIP2;7* might help in faster water uptake through outer water channels leading to faster imbibition thus accelerating germination even under normal conditions compared to control. In contrast, JcTIP1;3 which is internally localized to the vacuolar membrane probably functions more in maintaining cell turgidity and might interact more intricately with the cellular developmental and stress signalling machinery as has been reported for other TIPS ([3,43]. The higher expression and higher water uptake in the vacuole might activate GA responses and suppress ABA responses during germination as seen in the TIP expressing lines.

The effects of *JcPIP2;7* and *JcTIP1;3* expression were not restricted only to germination but were also seen in root growth under stressed conditions. Although transgenic lines expressing both aquaporins behaved similar to the control in absence of any stress, the application of osmotic stress led to a considerably higher reduction in root length in controls compared to those expressing the two aquaporins. Both dehydration and salt stresses are known to reduce root hydraulic conductivity and thereby inhibit root and plant growth [44–46]. The reduction in root hydraulic conductivity is largely brought about by a reduction in the level of aquaporins (primarily PIPs but also TIPs [43]; and is also correlated with a dynamic change in the post-translational modifications such as phosphorylation and amidation that affect aquaporin function ([47]. The higher levels of both JcPIP2;7 and JcTIP1;3 in over-expressing lines would serve to partly overcome the reduction in endogenous aquaporin levels during these stresses leading to higher water channelling in roots and leaves which in turn would help in maintaining growth above the controls under mild to moderate stresses. Simultaneously, subtle structural differences within residues of the two *Jatropha* aquaporins that might prevent post-translational inactivation and allow them to function under abiotic stresses also cannot be ruled out. The improved water channelling from root to leaves through expression of the *Jatropha* aquaporins would also explain the higher RWC in the leaves of the transgenic plants and the ability of the plants to withstand and survive the dehydration and salt stresses. In turn, this would translate into higher yields compared to controls. Under more severe stresses more drastic response measures would come into play negating the effect of aquaporin over-expression.

Although the two genes are localized to the plasma membrane and vacuolar membrane and must respond to different cues to exert their effects in these compartments, their over-expression seems to have similar effects on stress responsive growth suggesting that both play a role in maintaining cellular water homeostasis. The mechanism of their action would nevertheless have to be different given the differences in their structure and location and indeed the effects of their over-expression differently affect expression of genes involved in ABA responses. *JcPIP2;7* expressing lines do not show much of a change in expression of *RD22*, *RD29A* and *RD29B* —genes that are activated upon abiotic stresses primarily in an ABA dependent manner. On the other hand, lines expressing *JcTIP1;3* show 2–3 fold higher transcript levels of *RD29A* and *RD29B* compared to controls, both in absence and in presence of salt an observation similar to that seen upon expression of TaNIP which also causes salt tolerance (25). This suggests that *JcTIP1;3* expression not only improves water uptake but also simultaneously primes the plant for better stress responses through the activation of these genes. TIPs are known to respond more to abiotic stresses (which are in turn governed in an ABA dependent or independent manner) [25, 48–49] as evident from various studies such as on *TsTIP1;2*, tomato *SlTIP2;2* and *McTIP1;2* [6, 50–51] which respond to abiotic stresses and confer stress tolerance. Besides, certain TIPs have also been shown to function as H_2_O_2_ transporters and the possibility that *JcTIP1;3* might function as a ROS signaling transporter to activate stress responses through the ABA pathway cannot be discounted.

Finally, an important observation we have made was that although seed yields of plants grown on salt were much reduced compared to those not grown on salt, germination of seeds obtained from salt grown plants was surprisingly greatly improved in transgenic lines expressing *JcPIP2;7* and *JcTIP1;3*. Compared to just 3% germination of seeds obtained from salt grown control plants, the seeds of both the *Jatropha* aquaporin expressing plants grown on salt showed 45–50% viability i.e. about 10 folds higher viability than control. Thus the loss of viability seen in seeds of salt grown control plants was greatly reduced in plants expressing the two Jatropha aquaporins. This has important implications: as salinization of soils increases globally, a reduction in viability of seeds obtained from plants grown on such saline soils might turn out to be as big a problem as reduction in yields. The ability of *JcPIP2;7* and *JcTIP1;3* to overcome this can be a major application in agriculture and has so far not been reported to our knowledge.

## Conclusion

In conclusion we have identified two seed and water stress activated aquaporin genes *JcPIP2;7* and *JcTIP1;3* that upon heterologous expression in Arabidopsis improve plant growth under mild to moderate drought and salt stresses. Their expression improves root growth, RWC and survival under drought and salt stresses leading to higher seed yields. Importantly, seeds of transgenic lines expressing these aquaporins obtained from salt stress soils show ten folds higher viability compared to controls, an observation that has important implications for crops grown on saline soils.

## Supporting Information

S1 Fig Effect of Jatropha aquaporins expression on mutant yeast growth under salt stress. (PDF)

S2 Fig Percent germination of the wild type Col0 and transgenic Arabidopsis seeds expressing *JcPIP2;7* (A) and *JcTIP1;3* (B) in presence of ABA (1μM) after 48 h. (PDF)

S3 Fig Percent germination of the wild type Col0 and transgenic seeds in presence of NaCL and mannitol after 48 h. (PDF)

S4 Fig Effect of *JcPIP2;7* and *JcTIP1;3* over-expression on abiotic stress-related genes in transgenic Arabidopsis. (PDF)

S1 Table List of primers used in this study. (DOCX)

S2 Table Constructs used for yeast transformation. (DOCX)

## References

[1] Qadir M, Quillérou E, Nangia V, Murtaza G, Singh M, Thomas RJ, et al Economics of salt-induced land degradation and restoration. Natural Resources Forum 2014; 38: 282–295. 10.1111/1477-8947.12054

[2] Farquhar G, Sharkey T. Stomatal conductance and photosynthesis. Annu Rev Plant Physiol 1982; 33: 317–345.

[3] Cornic G. Drought stress inhibits photosynthesis by decreasing stomatal aperture—not by affecting ATP synthesis. Trends Plant Sci 2000; 5: 187–18.

[4] Chaves MM, Pereira JS, Maroco J, Rodrigues ML, Ricardo CPP, Osorio ML, et al How plants cope with water stress in the field? *Photosynthesis and growth* . Ann Bot (Lond) 2002; 89: 907–916. 10.1093/aob/mcf105 PMC4233809 12102516

[5] Chrispeels MJ, Maurel C. Aquaporins: the molecular basis of facilitated water movement through living plant cells. Plant Physiol. 1994;105: 9–15. 7518091 10.1104/pp.105.1.9 PMC159323

[6] Krich HH, Veera-Estrella R, Golldack D, Quigley F, Michalowski CB, Barkla DJ, Bohnert HJ. Expression of water channel proteins in *Mesibryanthemum crystallinum* Plant Physiol. 2000;123: 111–124. 10806230 10.1104/pp.123.1.111 PMC58987

[7] Tyerman S.D, Niemietz CM, Bramley H, Plant aquaporins: multifunctional water and solute channels with expanding roles. Plant Cell Environ. 2002;25: 173–194. 11841662 10.1046/j.0016-8025.2001.00791.x

[8] Hachez C, Zelazny E, Chaumont F. Modulating the expression of aquaporin genes in planta: a key to understand their physiological functions? Biochim Biophys Acta 2006;1758: 1142–1156. 16580626 10.1016/j.bbamem.2006.02.017

[9] Maurel C, Verdoucq L, Luu DT, and Santoni V. Plant Aquaporins: Membrane Channels with Multiple Integrated Functions. Annu Rev Plant Biol. 2008;59: 595–624. 10.1146/annurev.arplant.59.032607.092734 18444909

[10] Agre P. Clinical relevance of basic research on red cell membranes. Clin Res. 1992; 40:176–186. 1576797

[11] Chrispeels MJ, Agre P. Aquaporins: water channel proteins of plant and animal cells. Trends Biochem Sci. 1994;19: 421–425. 7529436 10.1016/0968-0004(94)90091-4

[12] Schaeffner AR. Aquaporin function, structure, and expression: are there more surprises to surface in water relations? Planta 1998; 204: 131–139. 9487723 10.1007/s004250050239

[13] Johanson U, Karlsson M, Johansson I, Gustavsson S, Sjovall S, Fraysse L, et al The complete set of genes encoding major intrinsic proteins in Arabidopsis provides a framework for a new nomenclature for major intrinsic proteins in plants. Plant Physiol. 2001;126(4): 1358–1369. 11500536 10.1104/pp.126.4.1358 PMC117137

[14] Quigley F, Rosenberg JM, Shachar-Hill Y, Bohnert HJ. From genome to function: the Arabidopsis aquaporins. Genome Biol. 2001;3: 1–17. 10.1186/gb-2001-3-1-research0001 PMC150448 11806824

[15] Sakurai J, Ishikawa F, Yamaguchi T, Uemura M, Maeshima M. Identification of 33 rice aquaporin genes and analysis of their expression and function. Plant Cell Physiol. 2005;46: 1568–1577. 16033806 10.1093/pcp/pci172

[16] Fouquet R, Leon C, Ollat N, Barrieu F. Identification of grapevine aquaporins and expression analysis in developing berries. Plant Cell Rep. 2008;27(9): 1541–1550. 10.1007/s00299-008-0566-1 18560835

[17] Park W, Scheffler BE, Bauer PJ, CampbeIl BT. Identification of the family of aquaporin genes and their expression in upland cotton (*Gossypium hirsutum L*.). BMC Plant Biol. 2010;10: 142–158. 10.1186/1471-2229-10-142 20626869 PMC3095289

[18] Ishikawa F, Suga S, Uemura T, Sato MH, Maeshima M. Novel type aquaporin SIPs are mainly localized to the ER membrane and show cell-specific expression in *Arabidopsis thaliana* . FEBS Lett. 2005;579: 5814–5820. 16223486 10.1016/j.febslet.2005.09.076

[19] Gomes D, Agasse A, Thiébaud P, Delrot S, Gerós H, Chaumont F. Aquaporins are multifunctional water and solute transporters highly divergent in living organisms. Biochim Biophys Acta 2009;1788: 1213–1228 10.1016/j.bbamem.2009.03.009 19327343

[20] Zhou Y, Setz N, NiemietzI C, Qu H, Offler CE, Tyerman, JW P Aquaporins and unloading of phloem-imported water in coats of developing bean seeds. Plant Cell and Environ 2007; 30: 1566–1577. 17927694 10.1111/j.1365-3040.2007.01732.x

[21] Smart LB, Moskal WA, Cameron KD, Bennett AB. MIP genes are down-regulated under drought stress in *Nicotiana glauca* . Plant and Cell Physiology 2001;42: 686–693. 11479374 10.1093/pcp/pce085

[22] Alexandersson E, Fraysse L, Sjovall-Larsen S, Gustavsson S, Fellert M, Karlsson M, et al Whole gene family expression and drought stress regulation of aquaporins. Plant Mol Biol. 2005; 59: 469–484. 16235111 10.1007/s11103-005-0352-1

[23] Zhu C, Schraut D, Hartung W, Schäffner AR. Differential responses of maize MIP genes to salt stress and ABA. J Exp Bot 2005; 56:2 971–2981. 10.1093/jxb/eri294 16216844

[24] Guo L, Wang ZY, Lin H, Cui WE, Chen J, Liu M. Expression and functional analysis of the rice plasma-membrane intrinsic protein gene family. Cell Res. 2006; 16: 277–286. 16541126 10.1038/sj.cr.7310035

[25] Gao Z, He X, Zhao B, Zhou C, Liang Y, Ge R, et al Over-expressing a putative aquaporin gene from wheat, TaNIP, enhances salt tolerance in transgenic Arabidopsis. Plant and Cell Physiology 2010; 51: 767–775. 10.1093/pcp/pcq036 20360019

[26] Misra A, Khan K, Niranjan A, Nath P, Sane VA. Over-expression of JcDGAT1 from Jatropha curcas increases seed oil levels and alters oil quality in transgenic *Arabidopsis thaliana* . Phytochemistry 2013; 96: 37–45. 24125179 10.1016/j.phytochem.2013.09.020

[27] Singh RK, Mishra A, Sane VA, Nath P. Isolation of high quality RNA from oilseeds of *Jatropha curcas* . J Plant Biochem Biotechnol. 2009;18: 77–81.

[28] Altschul SF, Gish W, Miller W, Myers EW, Lipman DJ. Basic local alignment search tool. J Mol Biol. 1990;215: 403–410. 2231712 10.1016/S0022-2836(05)80360-2

[29] Thompson JD, Gibson TJ, Plewniak F, Jeanmougin F, Higgins DG. The CLUSTAL_X windows interface: Flexible strategies for multiple sequence alignment aided by quality analysis tools. Nucleic Acids Res. 1997; 25: 4876–4882. 9396791 10.1093/nar/25.24.4876 PMC147148

[30] Tamura K, Stecher G, Peterson D, Filipski A, Kumar S. MEGA6: Molecular Evolutionary Genetics Analysis version 6.0. Molecular Biology and Evolution. 2013; 30: 2725–2729. 10.1093/molbev/mst197 24132122 PMC3840312

[31] Nelson BK 1, Cai X, Nebenführ A. A multicolored set of in vivo organelle markers for co-localization studies in Arabidopsis and other plants. Plant J. 2007;51(6): 1126–1136. 17666025 10.1111/j.1365-313X.2007.03212.x

[32] Clough SJ, Bent AF. Floral dip: a simplified method for Agrobacterium-mediated transformation of *Arabidopsis thaliana* . Plant J. 1998;16: 735–743. 10069079 10.1046/j.1365-313x.1998.00343.x

[33] Weigel D, Glazebrook J. Arabidopsis: A Laboratory Manual. Cold Spring Harbor Laboratory Press, Cold Spring Harbor, NY 2002.

[34] Sato S, Hirakawa H, Isobe S, Fukai E, Watanabe A, Kato M, et al, Sequence analysis of the genome of an oil-bearing tree, *Jatropha curcas L* . DNA Res. 2011;18: 65–76 10.1093/dnares/dsq030 21149391 PMC3041505

[35] Jang HY, Yang SW, Carlson JE, Ku YG, Ahn SJ. Two aquaporins of Jatropha are regulated differentially during drought stress and subsequent recovery. J Plant Physiol. 2013;170(11): 1028–1038. 10.1016/j.jplph.2013.03.001 23537705

[36] Han G, Wang M, Yuan F, Na S, Song J,; Wang B. The CCCH zinc finger protein gene AtZFP1 improves salt resistance in Arabidopsis thaliana. Plant Mol Biol 2014; 86:237–253. 10.1007/s11103-014-0226-5 25074582

[37] Kaldenhoff R, RibasCarbo M, Sans JF, Lovisolo C, Heckwolf M, Uehlein N. Aquaporins and plant water balance. Plant Cell Environ. 2008;31: 658–666. 10.1111/j.1365-3040.2008.01792.x 18266903

[38] Laur J, Hacke UG. The Role of Water Channel Proteins in Facilitating Recovery of Leaf Hydraulic Conductance from Water Stress in *Populus trichocarpa* . PLoS ONE. 2014;9(11): e111751 10.1371/journal.pone.0111751 25406088 PMC4236056

[39] Peng YH, Lin WL, Cai WM, Arora R. Overexpression of a Panax ginseng tonoplast aquaporin alters sat tolerance, drought tolerance and cold acclimation ability in transgenic Arabidopsis plants. Planta. 2007;226: 729–740. 17443343 10.1007/s00425-007-0520-4

[40] Gao YP, Young L, Bonham-Smith P, Gusta LV. Characterization and expression of plasma and tonoplast membrane aquaporins in primed seed of *Brassica napus* during germination under stress conditions. Plant Mol Biol. 1999; 40: 635–644. 10480387 10.1023/a:1006212216876

[41] Liu HY, Yu X, Cui DY, Sun MH, Sun WN. The role of water channel proteins and nitric oxide signaling in rice seed germination. Cell Res. 2007;17: 638–649. 17452999 10.1038/cr.2007.34

[42] Maurel C, Tacnet F, Guclu J, Guern J, Ripoche P. Purified vesicles of tobacco cell vacuolar and plasma membranes exhibit dramatically different water permeability and water channel activity, Proc. Natl. Acad Sci. U. S. A. 1997; 94: 7103–7108. 11038555 10.1073/pnas.94.13.7103 PMC21292

[43] Boursiac Y, Chen S, Luu DT, Sorieul M, van den Dries N, Maurel C. Early effects of salinity on water transport in Arabidopsis roots. Molecular and cellular features of aquaporin expression. Plant Physiol 2005;139: 790–805 16183846 10.1104/pp.105.065029 PMC1255996

[44] Azaizeh H, Gunse B, Steudle E. Effects of NaCl and CaCl2 on water transport across root cells of maize (*Zea mays* L.) seedlings. Plant Physiology 1992; 99: 886–894. 16669016 10.1104/pp.99.3.886 PMC1080560

[45] Maggio A, Joly RJ. Effects of mercuric chloride on the hydraulic conductivity of tomato root systems: evidence for a channel-mediated water pathway. Plant Physiology 1995;109:331–335 12228599 10.1104/pp.109.1.331 PMC157593

[46] Carvajal M, Martinez V, Alcaraz CF. Physiological function of water channels as affected by salinity in roots of paprika pepper. Physiologia Plantarum 1999; 105, 95–101.

[47] di Pietro M, Vialaret J, Li GW, Hem S, Prado K, Rossignol M, et al Coordinated post-translational responses of aquaporins to abiotic and nutritional stimuli in Arabidopsis roots. Mol Cell Proteomics 2013;12: 3886–3897 10.1074/mcp.M113.028241 24056735 PMC3861731

[48] Katsuhara M, Koshio K, Shibasaka M, Hayashi Y, Hayakawa T, Kasamo K. Over-expression of a barley aquapo*rin* increased the shoot/root ratio and raised salt sensitivity in transgenic rice plants. Plant Cell Physiol. 2003;44: 1378–1383. 14701933 10.1093/pcp/pcg167

[49] Lian HL, Yu X, Lane D, Sun WN, Tang ZC, Su WA. Upland rice and low land rice exhibited different PIP expression under water deficit and ABA treatment. Cell Res 2006; 16:651–60. 16773042 10.1038/sj.cr.7310068

[50] Wang LL, Chen AP, Zhong NQ, Liu N, Wu XM, Wang F, et al The Thellungiella salsuginea tonoplast aquaporin TsTIP1;2 functions in protection against multiple abiotic stresses. Plant Cell Physiol 2014; 55(1): 148–161. 10.1093/pcp/pct166 24214268 PMC3894706

[51] Wang Sade N, Vinocur BJ, Diber A, Shatil A, Ronen G, Nissan H, et al Improving plant stress tolerance and yield production: is the tonoplast aquaporin SlTIP2;2 a key to isohydric to anisohydric conversion? New Phytologist. 2009;181: 651–661. 10.1111/j.1469-8137.2008.02689.x 19054338

