# Supplementary material for: Heterologous Expression of Two Jatropha Aquaporins Imparts Drought and Salt Tolerance and Improves Seed Viability in Transgenic Arabidopsis thaliana
Source: PLoS One. 2015 Jun 12;10(6):e0128866. doi: 10.1371/journal.pone.0128866 (PMC4466373; doi:10.1371/journal.pone.0128866)

SFig 1. Effect of Jatropha aquaporins expression on mutant yeast growth under salt stress. (A, B**)** Strains expressing *JcPIP2;7* (VAS P2;7) and *JcTIP1;3* (VAS T1;3) and empty vector (VAS0) were grown in YNB medium till OD_{600} 1. Serially diluted culture in sterile water was spotted onto YNB plates containing 0, 75, 100mM NaCl. Images were taken after 5 days incubation at 28°C. Identical results were obtained in three independent experiments.

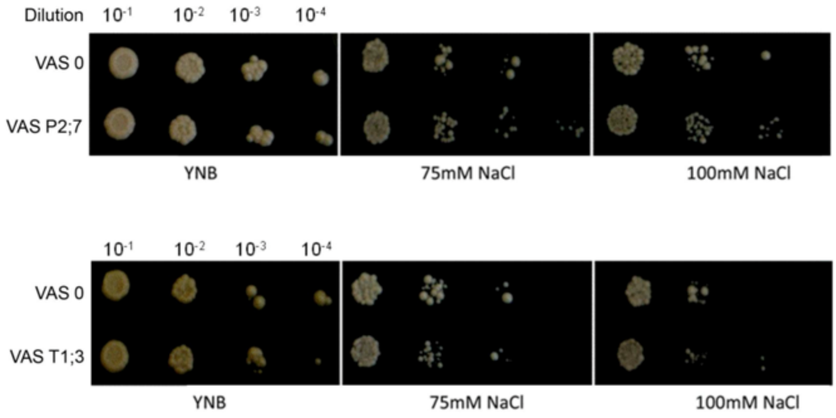

Supplement: S1 Fig — (PDF) [file pone.0128866.s001.pdf]
