# Supplementary material for: Heterologous Expression of Two Jatropha Aquaporins Imparts Drought and Salt Tolerance and Improves Seed Viability in Transgenic Arabidopsis thaliana
Source: PLoS One. 2015 Jun 12;10(6):e0128866. doi: 10.1371/journal.pone.0128866 (PMC4466373; doi:10.1371/journal.pone.0128866)
Supplement: S2 Fig — (PDF) [file pone.0128866.s002.pdf]

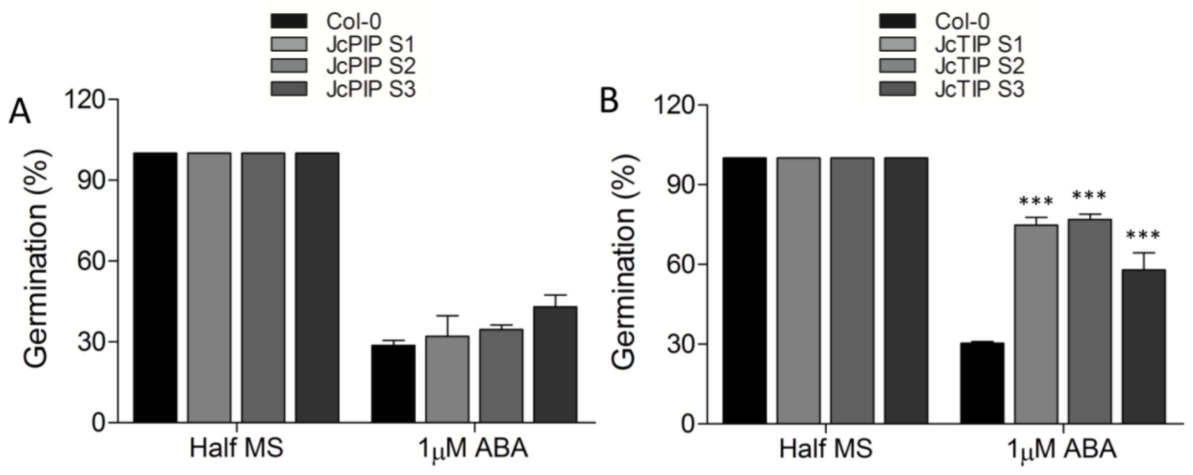

SFig 2: Germination (%) of the wild type Col0 and transgenic Arabidopsis seeds expressing *JcPIP2;7* **(A)** and *JcTIP1;3* **(B)** in presence of ABA (1µM) after 48 h . Values and means obtained from three independent experiments. Error bars represent ±SD, ****P*<0.001.
