# Supplementary material for: Heterologous Expression of Two Jatropha Aquaporins Imparts Drought and Salt Tolerance and Improves Seed Viability in Transgenic Arabidopsis thaliana
Source: PLoS One. 2015 Jun 12;10(6):e0128866. doi: 10.1371/journal.pone.0128866 (PMC4466373; doi:10.1371/journal.pone.0128866)
Supplement: S3 Fig — (PDF) [file pone.0128866.s003.pdf]

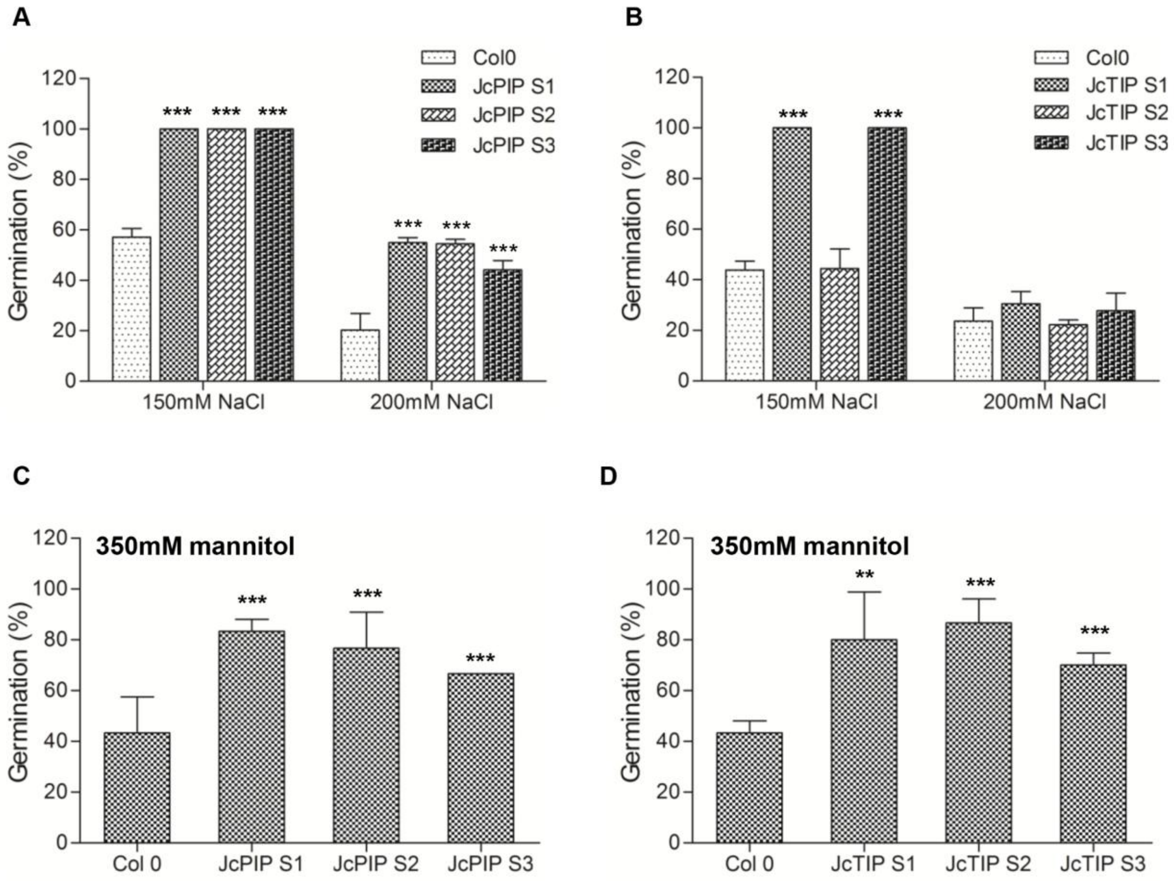

SFig 3: Germination (%) of the wild type Col0 and transgenic seeds was measured at day 2 on half MS medium supplemented with 150, 200mM NaCl **(**A, B**)** and 350mM mannitol **(**C, D**).**
