# Supplementary material for: Heterologous Expression of Two Jatropha Aquaporins Imparts Drought and Salt Tolerance and Improves Seed Viability in Transgenic Arabidopsis thaliana
Source: PLoS One. 2015 Jun 12;10(6):e0128866. doi: 10.1371/journal.pone.0128866 (PMC4466373; doi:10.1371/journal.pone.0128866)
Supplement: S4 Fig — (PDF) [file pone.0128866.s004.pdf]

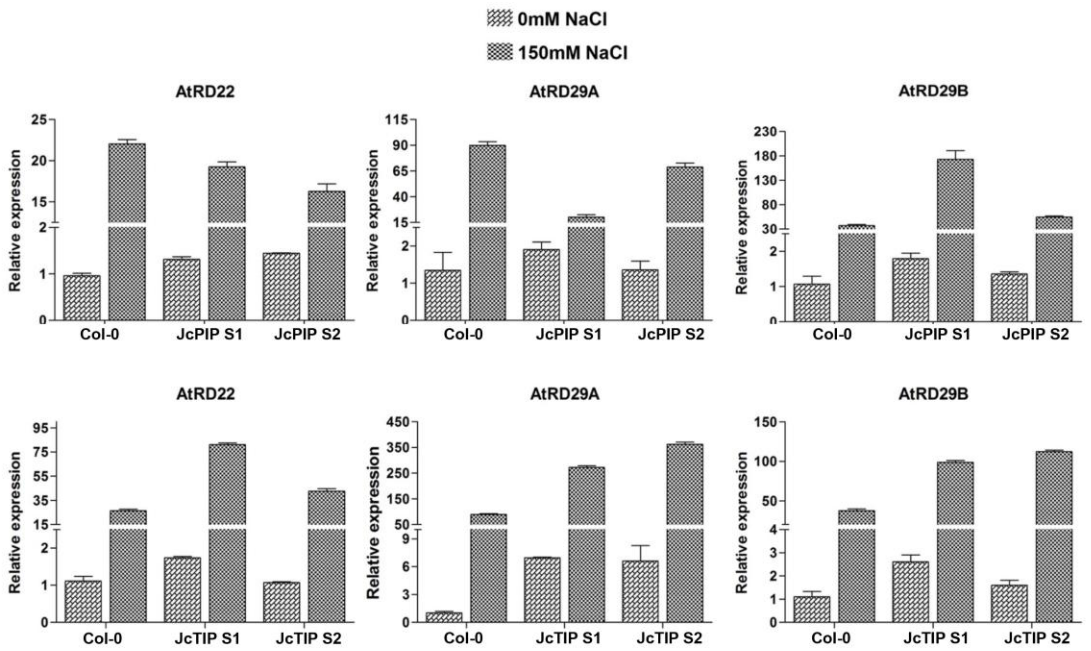

SFig 4: **Effect of *JcPIP2;7* and *JcTIP1;3* over-expression on abiotic stress-related genes in Arabidopsis**. Expression of AtRD22, AtRD29A, and AtRD29B in leaves of control and transgenic lines expressing *JcPIP2;7* (upper panel) and *JcTIP1;3 (lower panel)*. All the plants were grown on soilrite for 4 weeks with and without 150mM NaCl. Leaves were taken for RNA extraction and qRTPCR analysis. Values are the mean and error bars represent ±SD (n = *3).*
