# Supplementary material for: Heterologous Expression of Two Jatropha Aquaporins Imparts Drought and Salt Tolerance and Improves Seed Viability in Transgenic Arabidopsis thaliana
Source: PLoS One. 2015 Jun 12;10(6):e0128866. doi: 10.1371/journal.pone.0128866 (PMC4466373; doi:10.1371/journal.pone.0128866)
Supplement: S1 Table — (DOCX) [file pone.0128866.s005.docx]

**Khan et al., Supporting Information**

**STable 1. List of primers used in this study**

| JcTIPF0 | GACG*T*C*TA*G*A*ATGCCGATCAGAAAC |
| --- | --- |
| JcTIPR0 | GC*GA*G*C*T*C*TTAGTAGTCAGCGGTAG |
| JcTIPqF1 | GTCATTTGGACCAGCTTTGG |
| JcTIPqF2 | CTTGCTGGGCTCGTTTATGA |
| JcTIPqR1 | GATCATTCATCCCCAACCAC |
| JcTIPqR2 | AAGTTGCTCGTGGGTGTTGT |
| JcPIPFo | GTCTAGAATGGCCAAGGAAGTAAGTGAAG |
| JcPIPRo | TGAGCTCTTAGTTGGTGGGGTTGCTGC |
| JCPIPFq | CTGGGTTGGACCGTTTGTTGGA |
| CaMV35SF | GTAAGGGATGACGCACAATCC’ |
| NOST Rev | GGACTCTAATCATAAAAACCC |
| M13-REV | CAGGAAACAGCTATGACCATG |
| M13U | GTAAAACGACGGCCAGT |
| JcActinFq | GCCCCTGAGGAACACCCAGTGC |
| JcActinRq | GCAGGCACATTAAAGGTCTCAAAC |
