# Supplementary material for: Heterologous Expression of Two Jatropha Aquaporins Imparts Drought and Salt Tolerance and Improves Seed Viability in Transgenic Arabidopsis thaliana
Source: PLoS One. 2015 Jun 12;10(6):e0128866. doi: 10.1371/journal.pone.0128866 (PMC4466373; doi:10.1371/journal.pone.0128866)
Supplement: S2 Table — (DOCX) [file pone.0128866.s006.docx]

**Khan et al , Supporting information**

**STable 2 Constructs used for yeast transformation**

|  |  | Source |
| --- | --- | --- |
| **Empty plasmid** | pUG35 | U. Gu¨ ldener and J. H. Hegemann |
| ***S.cerevisae YSH1172*** | genotype 10560-6B MATa leu2::hisG trp1::hisG his3::hisG ura3-52 aqy1::KanMX4 aqy2::HIS3 P | Van Dijck |
| **VAS0** | YSH1172+ pUG35 | In this study |
| **VASP2;7** | YSH1172+ pUG35-JcPIP2;7 | In this study |
| **VAST1;3** | YSH1172+ pUG35-JcTIP1;3 | In this study |
